# Validation of the metabolic power model during three intermittent running-based exercises with emphasis on aerobic and anaerobic energy supply

**DOI:** 10.3389/fspor.2025.1583313

**Published:** 2025-04-17

**Authors:** Joana Brochhagen, Matthias W. Hoppe

**Affiliations:** ^1^ Exercise Science, Institute of Sport Science and Motology, Philipps University Marburg, Marburg, Germany; ^2^ Movement and Training Science, Faculty of Sport Science, Leipzig University, Leipzig, Germany

**Keywords:** energy cost, energy equivalent, external load, global positioning system, oxygen uptake kinetics, PCr-LA-O2 model

## Abstract

**Introduction:**

In intermittent sports, available internal load measurements like capillary blood techniques and portable respiratory gas analyzers are considered as gold standards in controlled laboratory environments, but are impractical for daily use in training and matches. A newer approach, the metabolic power model, allows to extrapolate from speed and acceleration data to the metabolic power, simulated oxygen uptake, and aerobic and anaerobic energy supply. The aim of this study was to validate the metabolic power model against the established 3-component model to allow direct comparison of variables including energy expenditure and supplies during intermittent running-based exercises.

**Methods:**

Twelve male athletes (24 ± 3 years) performed three different running-based exercises consisting of continuous shuttle runs and repeated accelerations and sprints with change of direction. Each exercise condition intended to primarily stress the aerobic, anaerobic alactic, and lactic energy supply. One-way repeated measures ANOVA or Friedman test and corresponding effect sizes were applied for statistical analyses. Additionally, absolute and relative biases and Bland-Altman plots were generated.

**Results:**

For total energy expenditure, there were statistically significant differences (*p* ≤ .002, *d* ≥ .882, large) and biases of −13.5 ± 11.8% for the continuous shuttle runs and up to 352.2 ± 115.9% for repeated accelerations and sprints. Concerning aerobic energy supply, there were statistically significant differences (*p* < .001, *d* ≥ 1.937, large effect sizes) and biases of up to −38.1 ± 11.7%. For anaerobic energy supply, there were statistically significant differences (*p* < .001, *d* ≥ 5.465, large) and biases of up to 1,849.9 ± 831.8%.

**Discussion:**

In conclusion, the metabolic power model significantly under- or overestimates total energy expenditure and supplies with large effect sizes during intermittent running-based exercises. Future studies should optimize the model before it can be used on a daily basis for scientific and practical purposes.

## Introduction

In intermittent sports, monitoring of external and internal loads has become an indispensable part of training and matches (1). Whereas the external load is defined as the conducted physical work of exercises, the internal load represents the resulting psychophysiological response, which is involved in the regulation of the gene expression and thus adaptation processes with respect to performance and health outcomes (2–4). It is well accepted that external loads such as total distances covered or times spent in different speed and acceleration categories can be measured in a valid and practical manner by tracking technologies including global and local positioning systems (5, 6). However, more complex activities like changes of direction, jumps, collisions, and technical skills (e.g., ball handling) are still a challenge when tracking external loads (7, 8), mainly due to the measurement principle of tracking technologies and their data processing approaches only allowing to quantify the *x*-/*y*-coordinates and derived variables of the center of mass in a valid manner (9). Importantly, with respect to internal (metabolic) load measurements such as capillary blood techniques and portable respiratory gas analyzers, which are still considered as gold standards in controlled laboratory environments, the available technological approaches may not be the best choices to investigate acute responses or long-term adaptations in intermittent sports. This is because they are partially invasive, poorly reproducible, and unsuitable for the use in daily training processes and competitive matches (4, 10–12). Thus, new technological solutions for investigating internal (metabolic) loads in intermittent sports are worth to develop.

A newer approach is the metabolic power model, which extrapolates from external (mechanical) to internal (metabolic) load (4, 13). This requires two basic assumptions: (i) the energy cost of accelerated running on a flat terrain is equivalent to running uphill at a constant speed and (ii) the relative energy cost for running is independent of speed and amounts to approximately 3.6–4.0 J/kg/m (14, 15). Through tracking technologies, the necessary inputs, namely speed and acceleration, can be validly and practicably accessed (6, 16). Then, the energy cost and, when multiplied by speed, instantaneous metabolic power can be estimated, whereby the latter is the amount of energy needed (displayed in W/kg) to maintain a constant ATP level (17). Additionally, once metabolic power is given, net oxygen uptake can be simulated by a physiological model and exponential function taking the time delay of approximately 20 s of the oxidative kinetics between the muscle and upper airway level into account. Then, it is possible to distinguish between aerobic and anaerobic energy supply, depending on the time courses of metabolic power and simulated oxygen uptake (18). Overall, the potential of the metabolic power model to investigate metabolic loads in intermittent sports is promising, especially since it offers instantaneous metabolic data during the entire training or match including the identification of crucial high-intensity activities (e.g., accelerations) (4). Such knowledge may help to optimize exercise-induced adaptation processes concerning performance and health outcomes. However, further research is necessary before a routine implementation of the metabolic power model into science and practice can be considered.

During the last years, several validation studies on the metabolic power model in intermittent sports were conducted and summarized within a recently published systematic review (19). The included studies mostly investigated professional male soccer players during official matches (20–23), small-sided games (24–26), sport-specific circuits (27–29), and constant and shuttle running exercises (22, 27, 29, 30). The metabolic power model was validated against established gold standards for measuring metabolic loads, mostly oxygen uptake collected by portable respiratory gas analyzers (28, 29, 31, 32). By the systematic review, strong evidence was shown that the energy expenditure during team-sport-specific circuits including activities such as multidirectional running, jumps, collisions, and technical skills assessed by the metabolic power model is 29%–52% lower when compared to oxygen uptake (19). However, in many of the reviewed validation studies, activities were conducted, which cannot be registered by the metabolic power model due to the measurement approach by tracking technologies, in particular, jumps, collisions, and technical skills such as ball handling (28, 32). Additionally, many studies included passive breaks (27–29, 31, 32), in which no data was generated by tracking technologies, whereas oxygen uptake was still elevated (18). Furthermore, it is important to consider that oxygen uptake only discloses aerobic energy supply during intermittent exercises, whereas metabolic power contains both aerobic as well as anaerobic alactic and lactic energy supply (17), which was unfortunately overlooked. Taken together, there is strong evidence for an underestimated energy expenditure by the metabolic power model; however, the inaccurate inclusion of certain activities, e.g., collisions or ball handling, and passive breaks as well as the exclusion of anaerobic energy supply should be considered (19). Consequently, there is a need for further validation studies eliminating these flaws, which may also allow an optimization of the model in the future.

The aim of this study was to validate the metabolic power model against an established approach of aerobic as well as anaerobic alactic and lactic energy supply to allow direct comparison of variables including energy expenditure and supplies during intermittent running-based exercises.

## Material and methods

### Participants

Twelve male trained athletes (24 ± 3 years; 185.0 ± 9.1 cm; 81.2 ± 11.1 kg) participated. They were recruited from the sports students of the local university. The recruitment period started on June 20th 2022 and ended on August 12th 2022. The most played intermittent sport was soccer (*n* = 7), followed by field and ice hockey (*n* = 2), handball (*n* = 1), volleyball (*n* = 1), and tennis (*n* = 1). Maximum oxygen uptake and heart rate were 64.8 ± 4.9 ml/kg/min and 195 ± 12 1/min, respectively. Inclusion criteria were males with an age of 18–30, who participated three to four times per week in their intermittent sport. Exclusion criteria were acute or chronic diseases and injuries (i.e., respiratory infections or musculoskeletal issues) speaking against maximum testing. The activity level of the athletes was defined according to the Participant Classification Framework (33). All athletes signed a written informed consent. The study was approved by the Local Ethics Committee (2022.02.23_eb_136) and conducted in accordance to the Declaration of Helsinki.

### Study design

This cross-sectional study was performed outdoors on a regular tartan track. The time period of testing ran from July 19th to September 13th 2022, with a temperature of 26.8 ± 5.7°C, wind speed of 0.6 ± 0.3 m/s, and air humidity of 50.7 ± 18.5%. The athletes had to participate on four days, separated by one week each. On the first day, body height and mass were collected using a stadiometer (HR001, Tanita Europe BV, Amsterdam, Netherlands) and a digital scale (BC-601, Tanita Europe BV, Amsterdam, Netherlands), respectively. Additionally, oxygen uptake was measured for 10 min in a seated position to account for resting oxygen uptake (34). Afterward, the athletes performed an Interval Shuttle Run Test until exhaustion to determine maximum oxygen uptake. This test was chosen, because it allows a valid and reliable assessment of maximum oxygen uptake in a sport-specific manner (35, 36). The test was conducted as described in detail elsewhere (36). During the test, oxygen uptake was directly measured breath-by-breath by a portable respiratory gas analyzer (Cortex Medical, MetaMax 3B, Leipzig, Germany). To clarify exhaustion, athletes had to reach two out of the following three criteria (37, 38): (i) ≥95% of age-predicted maximum heart rate (220—age), (ii) blood lactate ≥8 mmol/L, and (iii) rating of perceived exertion ≥19, which was achieved in all included athletes. On the second, third, and fourth day, three different running-based exercises were conducted in randomized order. Before each exercise, a 10 min standardized warm-up was performed. The warm-up consisted of a series of progressively increasing ABC running exercises (e.g., ankle drill, high knee skip, butt kicker) and five submaximal accelerations at the end to prepare for the subsequent exercises. During the three exercises, the metabolic power model (13) and, as an established comparative standard, the 3-component model (also called PCr-LA-O2 model) (39) were applied simultaneously. For validity purposes, the following variables from both models were used: (i) total energy expenditure (W_TOT_) as the sum of (ii) aerobic (W_AER_) and (iii) anaerobic energy supply (W_ANA_). Since the metabolic power model cannot distinguish between both anaerobic pathways yet, W_ANA_ was considered as the sum of the alactic and lactic supply. Furthermore, to guarantee better comparison between both models and to control for the possible bias caused by elevated oxygen uptake during passive breaks in intermittent exercises, the corresponding aerobic supply between each effort was also calculated and subtracted from total aerobic supply. For descriptive purposes, the respective values when including the breaks are still given, however, in the further course of the study, only the values when excluding the breaks are taken into account to allow comparison. Additionally, as the only directly comparable variable of both models, the accumulated measured (3-component model) and simulated oxygen uptakes (metabolic power model) were compared. In all cases, net oxygen uptake was considered. Finally, to give more insight into the differences of the three running-based exercises, external, namely speed, acceleration, and deceleration as well as internal load variables, namely metabolic power, heart rate, blood lactate, and rating of perceived exertion were also displayed.

### Running-based exercises

**Figure 1:**
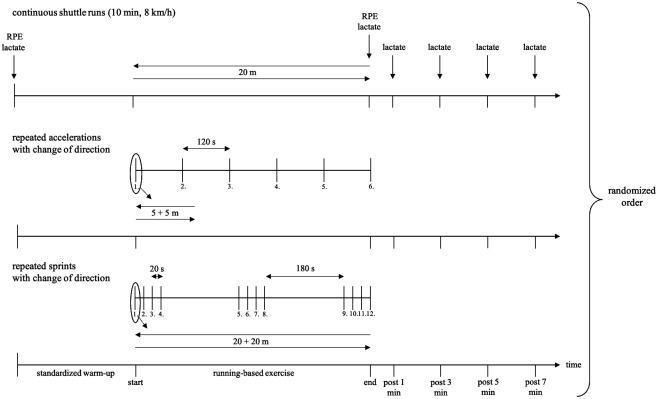
Design of the three running-based exercises. min, minute; RPE, rating of perceived exertion.

To question, if the potential biases concerning the metabolic power model are dependent on the type of energy supply, three running-based exercises were performed. Each exercise condition intended to primarily stress either the aerobic, anaerobic alactic or lactic energy supply. The three exercises were labeled as “continuous shuttle runs”, “repeated accelerations with change of direction”, and “repeated sprints with change of direction” (Figure 1), respectively. For the latter, the established repeated-sprint ability test by Rampinini et al. (40) was applied. The repeated sprints consisted of 3 × 4 × 20 + 20 m with a 180° change of direction and a 20 s or 3 min recovery after each sprint or after the fourth and eight sprint, respectively. In accordance with this, the continuous shuttle runs were carried out over 20 m with a constant speed at 8 km/h for 10 min. Concerning the repeated accelerations, the duration of the effort was chosen relatively short, given that anaerobic alactic energy supply can only be delivered for about 3–15 s during maximum load (41). Additionally, it is known that half time and full resynthesis of phosphocreatine is about 30 s and 2–4 min, respectively (42). Hence, recovery time was considered in this range. Based on these associations, the repeated accelerations consisted of 6 × 5 + 5 m runs with a 180° change of direction and 2 min recoveries. To ensure a time-normalized comparison, the number of repetitions for the sprints and accelerations was chosen as reported to achieve approximately the same duration of 10 min as for the continuous shuttle runs.

### Metabolic power model

Regarding the metabolic power model, a 20 Hz global positioning system (exelio srl, GPEXE LT, Udine, Italy) was worn in a vest between the shoulder blades. The typical error of the device was reported as 2.3% for speed and up to 5.6% for acceleration measures (16). Both variables are used as the calculation basis for the metabolic power model (4, 13) and the error rates are within those for commonly used metabolic carts ranging from 1.1%–13.3% for oxygen uptake, with 1.6% for the MetaMax 3B used in this study (43). The device was activated at least 5 min before each session to ensure optimal signal reception. During the data collection, the mean number of connected satellites and horizontal dilution of precision were 9 ± 1 and 1.0 ± 0.1, respectively, indicating ideal measurement conditions (44). The global positioning system provided the speed, from which the proprietary software then calculated the acceleration, metabolic power (Equations 1, 2), and simulated net oxygen uptake (Equation 3). The metabolic power was estimated via multiplying the energy cost by speed. The calculation details can be found in the original publications (4, 18); briefly, the used equations were as follows:

(1) $$\mathrm{EC}=(155.4\;ES^{5}-30.4\;ES^{4}-43.3\;ES^{3}+46.3\;ES^{2}+19.5\;\mathrm{ES}+3.6)\cdot \mathrm{EM},$$

where EC is the energy cost in J/kg/m, ES the equivalent slope gathered from the angle α of the athlete's body to the surface [=tan(90-α)], 3.6 the relative energy cost for running at constant speed in J/kg/m, and EM the equivalent mass as the force overload on the athlete from the acceleration (=g′/g).

**Figure 2:**
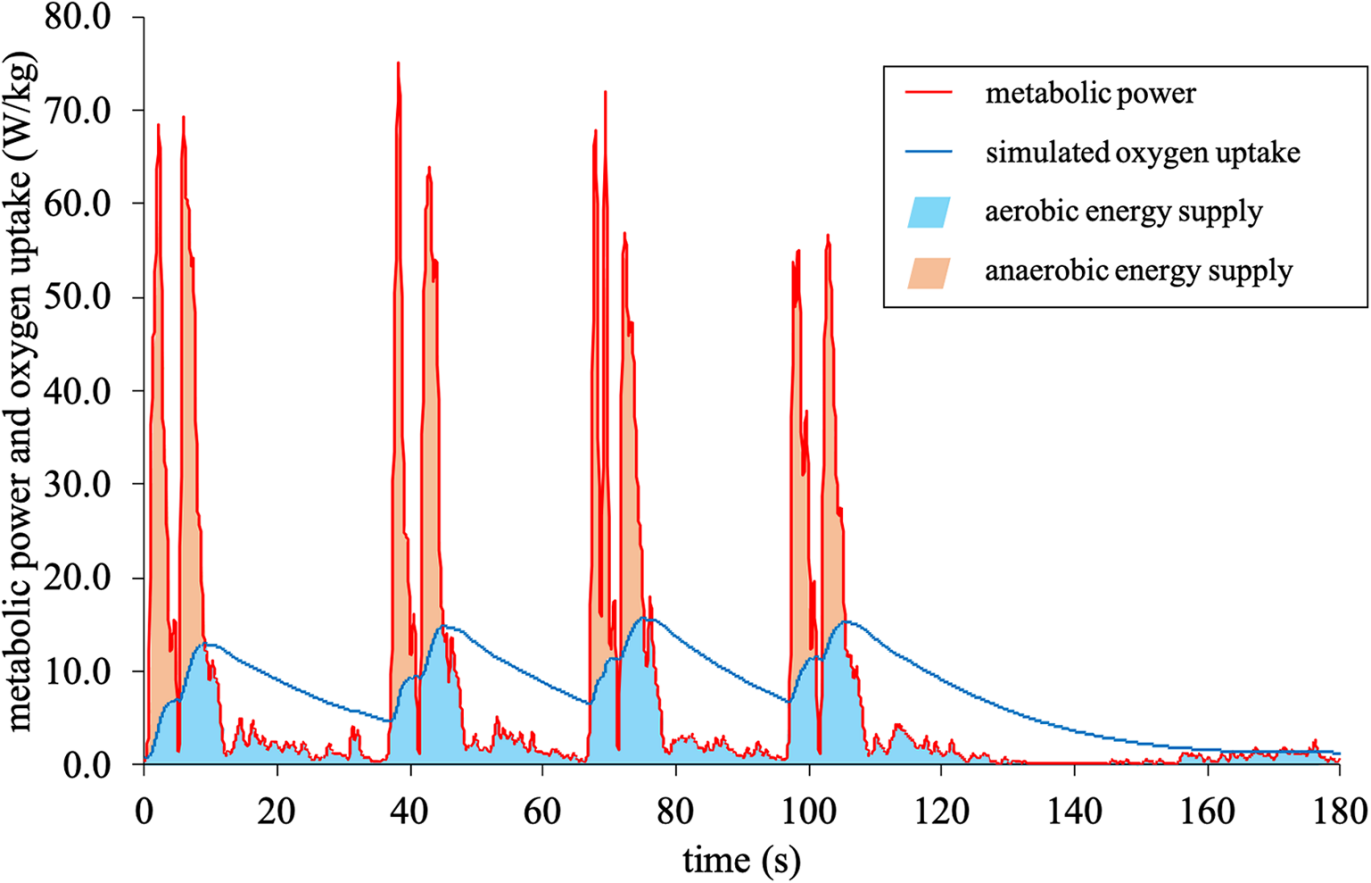
Metabolic power model: calculation of the aerobic and anaerobic energy supply.

(2) P=EC⋅v,

where *P* is the metabolic power in W/kg, EC is the energy cost in J/kg/m, and v the speed in m/s.

(3) $$\dot{V}O_{2}T_{n(t)}=({\dot{E}}_{n}-\dot{V}O_{2}T_{n(0)})\cdot \left(1-e^{-\frac{t}{\tau }}\right)+\dot{V}O_{2}T_{\left(0\right)},$$

where $\dot{V}O_{2}T_{n(t)}$ is the theoretical oxygen uptake at time t of each metabolic power interval, $\dot{V}O_{2}T_{n(0)}$ the theoretical oxygen uptake at the respective onset of each interval, $\dot{E}$ the metabolic power in equivalent oxygen uptake units, and τ the time constant. The maximum oxygen uptake, measured during the Interval Shuttle Run Test, was used as a cut-off criterion for the simulated oxygen uptake (18). The aerobic energy supply was computed as the time integral below the course of the simulated oxygen uptake while simultaneously being below the course of the metabolic power (18). Correspondingly, the anaerobic energy supply was calculated from the time integral below the course of the metabolic power, but above simulated oxygen uptake (18) (Figure 2). For these estimations, a fixed energy equivalent of 20.9 kJ/L O_2_ was assumed.

### 3-component model

**Figure 3:**
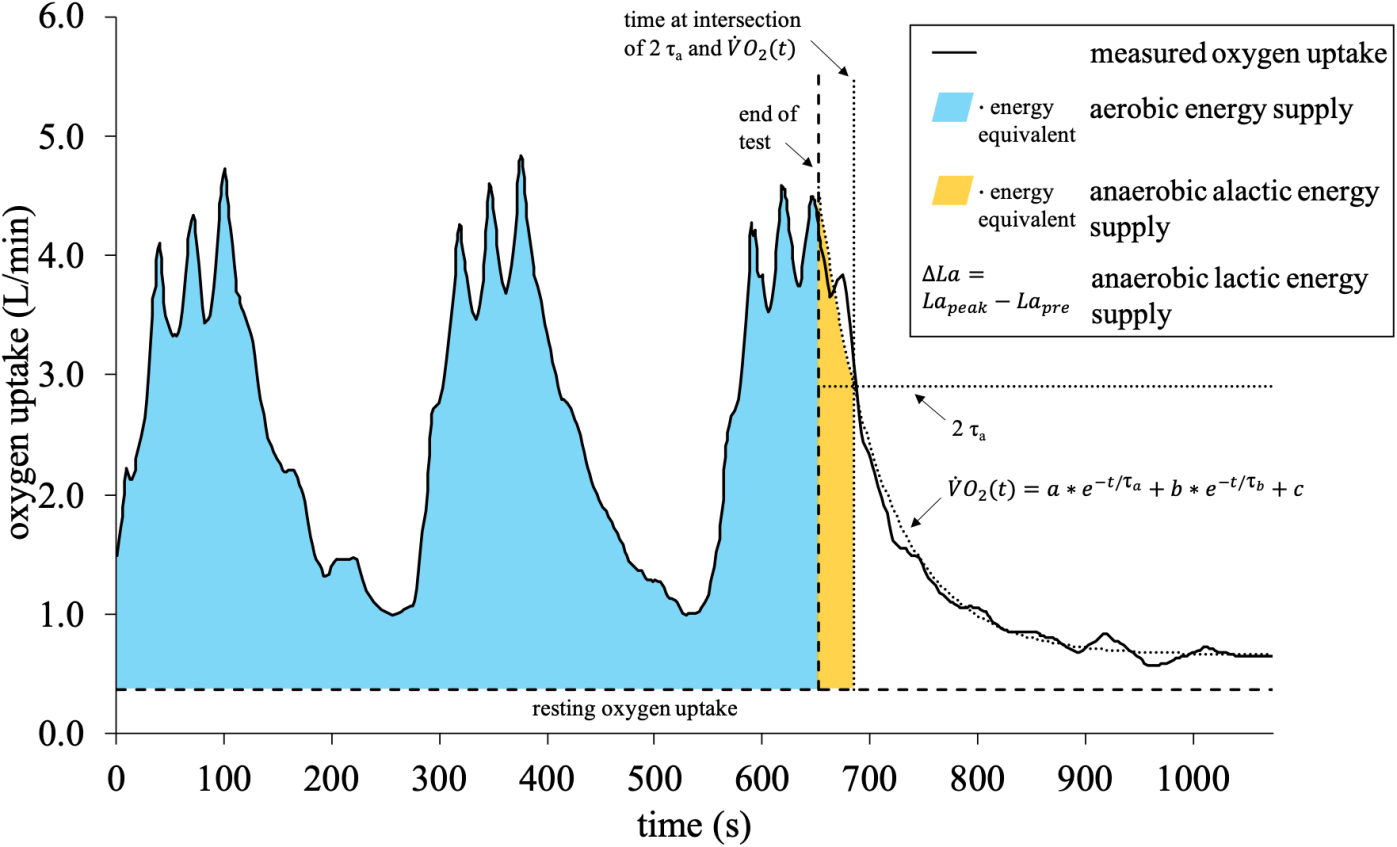
3-component model: calculation of the aerobic, anaerobic alactic, and lactic energy supply. $\dot{V}O_{2}(t)$ is the oxygen uptake at time *t*, *a* is the amplitude of the fast component, *b* is the amplitude of the slow component, $\tau_{a}$ and $\tau_{b}$ are the corresponding time constants, and *c* is the resting oxygen uptake.

Concerning the 3-component model, oxygen uptake was measured breath-by-breath using a portable respiratory gas analyzer (Cortex Medical, MetaMax 3B, Leipzig, Germany), which was calibrated prior to each data collection according to the manufacturers' instructions. The relative error of the MetaMax 3B for measuring oxygen uptake was reported as 1.6 ± 1.9% (43). For data processing, a moving average of ten breaths was applied, as reported before (45, 46). The method to distinguish between the three main energy supplies was carried out as described by Beneke et al. (39), which has been applied before (34, 47, 48). For all exercises, the net aerobic energy supply was calculated by multiplying the duration of the exercise by the resting oxygen uptake and subtracting the product from the product of accumulated oxygen uptake and the energy equivalent of oxygen (34, 39) (Figure 3). The anaerobic alactic energy supply was estimated from the fast component of excess post-exercise oxygen consumption, for which oxygen uptake was measured after every exercise for an additional 7 min in a seated position (34). The post-exercise oxygen consumption was fitted by a biexponential function shown in Figure 3 (39). The fast component was then defined until the predefined cut-off at 2 τ_a_ was reached. Finally, the corresponding time integral was multiplied by the energy equivalent of oxygen (34, 39). Regarding the energy equivalents, individual values based on the highest respiratory exchange ratio during each exercise were used as introduced by Zuntz and Schumburg (49). Additionally, to guarantee closer comparison, a fixed value of 20.9 kJ/L O_2_, as assumed by the metabolic power model, was applied. To determine the anaerobic lactic energy supply, 20 *µ*l capillary blood samples were taken from the right earlobe before and during the 1st, 3rd, 5th, and 7th minute after each exercise. The samples were collected in EDTA-coated capillaries, added into tubes with 1 ml hemolysis solution, and then analyzed by an electro-enzymatic analyzer (EKF-diagnostics, Biosen C_line Sport, Cardiff, United Kingdom) for the lactate concentration. Then, the Δ lactate was calculated by subtracting resting lactate from the highest post-exercise lactate. From the Δ lactate, the anaerobic lactic energy supply was computed, considering 1 mmol/L to be equivalent to 3 ml O_2_/kg (34, 50).

### Statistical analysis

For statistical analysis, all data were presented as means ± standard deviations and checked for normal distribution by the Shapiro–Wilk and variance homogeneity by Levene's test. Depending on the results for each variable individually, if normal distribution and variance homogeneity were given, a one-way repeated measures ANOVA and otherwise Friedman test was applied to compare global differences in means. The level of statistical significance was set to *p* < .05. Corresponding global effect sizes were calculated using generalized eta-squared ($\eta_{g}^{2}$) or Kendalls' W, being interpreted as small (≥.01; <.3), moderate (≥.059; <.5), and large (≥.138; ≥.5), respectively (51). According to normal distribution and variance homogeneity, *post-hoc* differences in means were computed using t-test or Wilcoxon test, taking Bonferroni correction into account. For pairwise effect sizes, Cohen's d was calculated and interpreted as follows: <.2 trivial, ≥.2 small, ≥.5 moderate, and ≥.8 large (51). Finally, absolute and relative biases (percentage differences between both models) and Bland-Altman plots with 95% limits of agreement were generated to display systematic differences and heteroscedasticities between measurements of both models.

## Results

**Figure 4:**
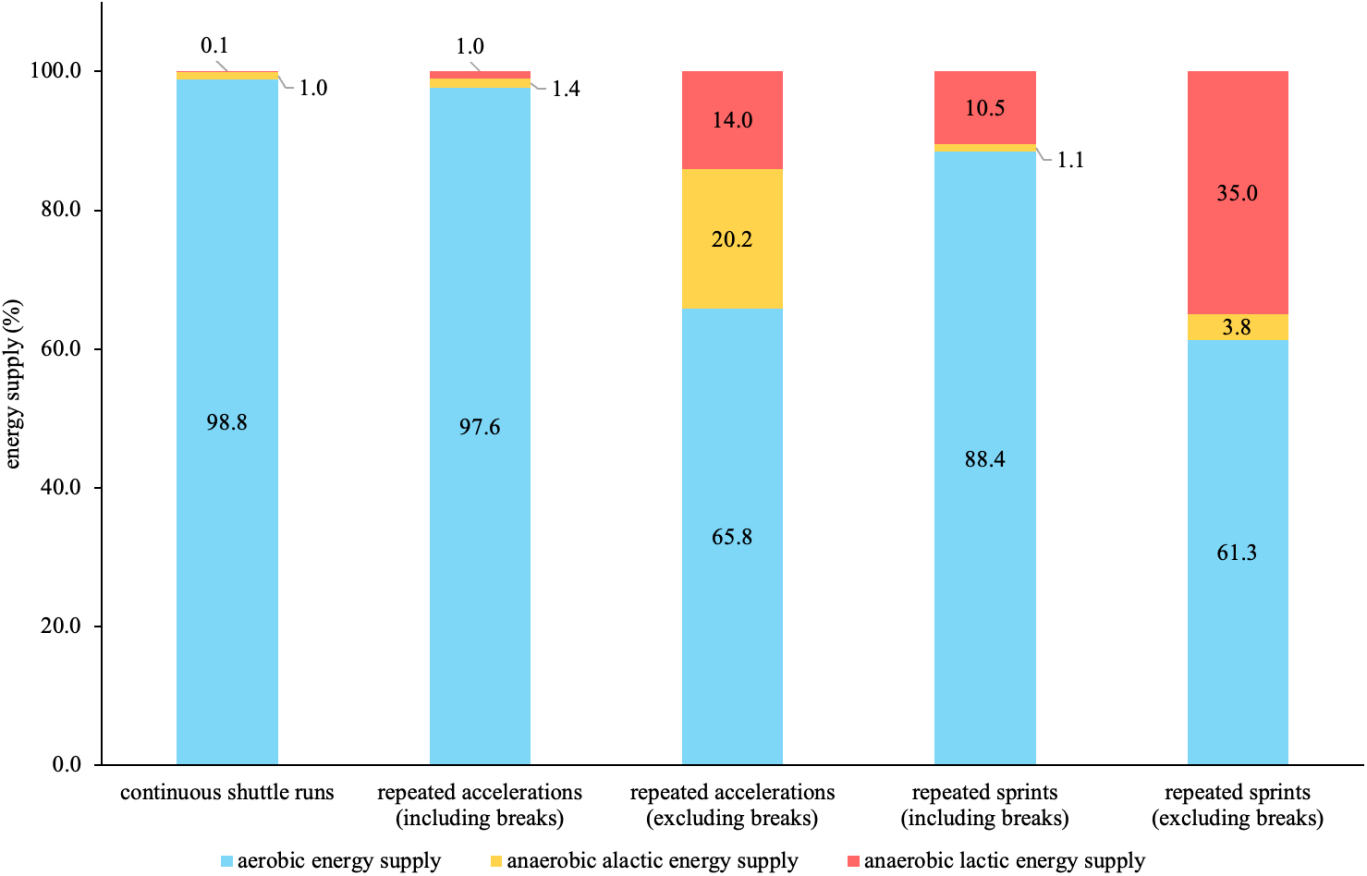
Relative energy supplies based on the 3-component model of the three running-based exercises.

Figure 4 shows the relative energy supplies based on the 3-component model of the three running-based exercises including and excluding the passive breaks. Regarding the continuous shuttle runs, aerobic supply was highest at 98.8%. When excluding the breaks from the other two exercises, aerobic supply was highest with 65.8%, followed by alactic with 20.2% and lactic supply with 14.0% for the repeated accelerations. The repeated sprints showed that aerobic supply was highest with 61.3%, followed by lactic with 35.0% and alactic supply with 3.8%.

Table 1 presents the differences in external and internal load measures during the three running-based exercises. For all variables, there were statistically significant differences (*p* ≤ .034, *d* ≥ .502, moderate to large). Exceptions were pre-exercise lactate and peak rating of perceived exertion for all differences (*p* ≥ .249, *d* ≤ .297, trivial to small) and between continuous shuttle runs and repeated accelerations (*p* = .797, *d* = .082, trivial), respectively.

**Table 1:** Differences in external and internal load measures during the three running-based exercises.

Variables		Continuous shuttle runs mean ± SD	Repeated accelerations with COD mean ± SD	Repeated sprints with COD mean ± SD	Global	C vs. RA	C vs. RS	RA vs. RS
					*p*-value	*p*-value	*p*-value	*p*-value
V_peak_ (m/s)^b^		3.50 ± 0.34	5.29 ± 0.55	7.19 ± 0.41	<.001^large^	<.001^large^	<.001^large^	<.001^large^
ACC_peak_ (m/s^2^)^a^		2.31 ± 0.29	4.02 ± 0.24	4.25 ± 0.29	<.001^large^	<.001^large^	<.001^large^	.015^large^
DEC_peak_ (m/s^2^)^b^		−2.07 ± 0.27	−3.09 ± 0.21	−4.94 ± 0.22	<.001^large^	<.001^large^	.003^large^	<.001^large^
Pmet_peak_ (W/kg)^a^		31.51 ± 5.72	75.33 ± 8.66	95.16 ± 11.74	<.001^large^	<.001^large^	<.001^large^	<.001^large^
HR_peak_ (1/min)^a^		149 ± 10	135 ± 9	180 ± 9	<.001^large^	<.001^large^	<.001^large^	<.001^large^
La_pre_ (mmol/L)^a^		1.62 ± 0.28	1.70 ± 0.35	1.60 ± 0.30	.490^small^	.249^small^	.881^trivial^	.352^small^
La_peak_ (mmol/L)^b^		1.74 ± 0.26	2.07 ± 0.42	13.73 ± 2.70	<.001^large^	.013^large^	<.001^large^	<.001^large^
ΔLa (mmol/L)^b^		0.12 ± 0.27	0.37 ± 0.22	12.13 ± 2.68	<.001^large^	.021^large^	<.001^large^	<.001^large^
RPE_peak_ (6–20)^b^		11 ± 2	11 ± 2	18 ± 1	<.001^large^	.797^trivial^	.002^large^	.002^large^
MPM	VO_2peak_ (ml/min/kg)^a^	28.9 ± 3.7	18.8 ± 2.5	52.2 ± 4.1	<.001^large^	<.001^large^	<.001^large^	<.001^large^
	VO_2mean_ (ml/min/kg)^b^	23.9 ± 2.7	4.9 ± 0.9	19.2 ± 1.2	<.001^large^	<.001^large^	<.001^large^	.003^large^
	VO_2accumulated_ (L)^b^	19.5 ± 3.6	4.2 ± 1.2	16.6 ± 2.6	<.001^large^	<.001^large^	<.001^large^	<.001^large^
3-CM	VO_2peak_ (ml/min/kg)^a^	36.1 ± 2.5	26.1 ± 5.2	62.5 ± 9.4	<.001^large^	<.001^large^	<.001^large^	<.001^large^
	VO_2mean_ (ml/min/kg)^a^	28.3 ± 2.0	10.8 ± 2.1	31.9 ± 1.9	<.001^large^	<.001^large^	.003^large^	<.001^large^
	VO_2accumulated_ (L)^b^	23.1 ± 3.6	9.0 ± 3.0	24.8 ± 3.1	<.001^large^	<.001^large^	.034^moderate^	<.001^large^

Means, standard deviations, *p*-values, and interpretations of the effect sizes are shown.

3-CM, 3-component model; ACC, acceleration; C, continuous shuttle runs; COD, change of direction; DEC, deceleration; HR, heart rate; La, lactate; MPM, metabolic power model; Pmet, metabolic power; RA, repeated accelerations with change of direction; RPE, rating of perceived exertion; RS, repeated sprints with change of direction; SD, standard deviation; V, speed; VO_2_, net oxygen consumption; ΔLa, net lactate concentration (La_peak_—La_pre_).

^a^ analyzed by one-way repeated measures ANOVA and *t*-test.

^b^ analyzed by Friedman and Wilcoxon test.

**Figure 5:**
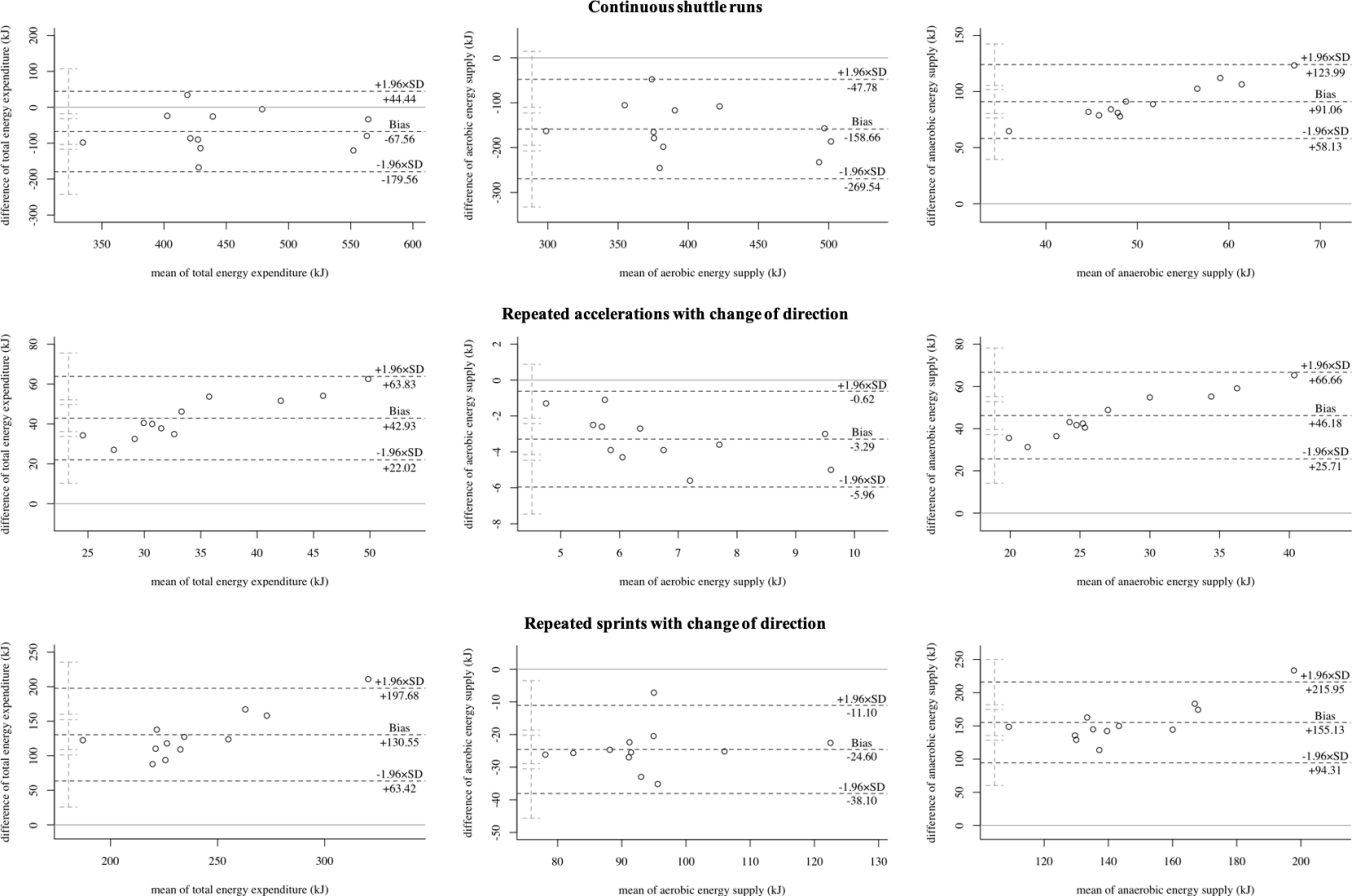
Bland-Altman plots of the total energy expenditure, aerobic, and anaerobic energy supply of the three running-based exercises. SD, standard deviation.

Table 2 displays the differences in the total energy expenditure and energy supplies during the three running-based exercises. Figure 5 shows the corresponding Bland-Altman plots displaying the systematic differences and heteroscedasticities between measurements of both models. Regarding total energy expenditure, there were statistically significant differences (*p* ≤ .002, *d* ≥ .882, large). The mean relative biases for the metabolic power model were −13.5 ± 11.8%, 352.2 ± 115.9%, and 75.0 ± 17.0% for the continuous shuttle runs, repeated accelerations, and repeated sprints, respectively. Concerning the aerobic energy supply, there were statistically significant differences (*p* < .001, *d* ≥ 1.937, large). The mean relative biases for the metabolic power model were −32.6 ± 10.3%, −38.1 ± 11.7%, and −23.2 ± 6.6% for the continuous shuttle runs, repeated accelerations, and repeated sprints, respectively. Also, for the anaerobic energy supply, there were statistically significant differences (*p* < .001, *d* ≥ 5.465, large). The mean relative biases for the metabolic power model were 1,849.9 ± 831.8%, 1,171.3 ± 514.1%, and 238.9 ± 75.8% for the continuous shuttle runs, repeated accelerations, and repeated sprints, respectively. Comparison between the usage of individual and fixed energy equivalents for the 3-component model showed statistically significant differences (*p* ≤ .031, *d* ≤ .102, trivial).

**Table 2:** Differences in total energy expenditure and energy supplies of the three running-based exercises using the metabolic power model and the 3-component model (individual and fixed energy equivalents).

Running-based exercises		Variables	Metabolic power model	3-component model		Global	MPM vs. ind	MPM vs. fix	ind vs. fix
				ind	fix	*p*-value	*p*-value	*p*-value	*p*-value
			mean ± SD	mean ± SD	mean ± SD				
Continuous shuttle runs		W_TOT_ (kJ)^a^	421.1 ± 77.2	491.7 ± 75.6	488.7 ± 76.1	.002^large^	.002^large^	.002^large^	.011^trivial^
		W_AER_ (kJ)^a^	324.4 ± 61.8	486.0 ± 75.2	483.0 ± 75.7	<.001^large^	<.001^large^	<.001^large^	.011^trivial^
		W_ANA_ (kJ)^b^	96.7 ± 16.8	5.7 ± 2.1	5.6 ± 2.1	<.001^large^	<.001^large^	<.001^large^	.031^trivial^
Repeated sprints with COD	Incl. breaks	W_TOT_ (kJ)^b^	92.6 ± 25.4	195.5 ± 64.0	193.4 ± 63.3	<.001^large^	<.001^large^	<.001^large^	<.001^trivial^
		W_AER_ (kJ)^b^	41.8 ± 15.1	190.9 ± 62.5	188.8 ± 61.8	<.001^large^	<.001^large^	<.001^large^	<.001^trivial^
		W_ANA_ (kJ)^b^	50.8 ± 11.4	4.6 ± 1.9	4.6 ± 1.9	<.001^large^	<.001^large^	<.001^large^	.002^trivial^
	Excl. breaks	W_TOT_ (kJ)^b^	55.9 ± 12.7	13.1 ± 3.8	12.9 ± 3.7	<.001^large^	<.001^large^	<.001^large^	.002^trivial^
		W_AER_ (kJ)^b^	5.1 ± 1.3	8.5 ± 2.0	8.4 ± 2.0	<.001^large^	<.001^large^	<.001^large^	.003^trivial^
		W_ANA_ (kJ)^b^	50.8 ± 11.4	4.6 ± 1.9	4.6 ± 1.9	<.001^large^	<.001^large^	<.001^large^	.002^trivial^
Repeated sprints with COD	Incl. breaks	W_TOT_ (kJ)^a^	376.1 ± 62.1	593.1 ± 75.1	586.6 ± 74.2	<.001^large^	<.001^large^	<.001^large^	<.001^trivial^
		W_AER_ (kJ)^a^	152.7 ± 26.5	524.1 ± 65.4	518.3 ± 64.7	<.001^large^	<.001^large^	<.001^large^	<.001^trivial^
		W_ANA_ (kJ)^a^	223.4 ± 37.1	69.0 ± 14.9	68.3 ± 14.7	<.001^large^	<.001^large^	<.001^large^	<.001^trivial^
	Excl. breaks	W_TOT_ (kJ)^a^	305.2 ± 49.0	176.6 ± 22.4	174.7 ± 22.1	<.001^large^	<.001^large^	<.001^large^	<.001^trivial^
		W_AER_ (kJ)^a^	81.8 ± 12.2	107.6 ± 11.5	106.4 ± 11.4	<.001^large^	<.001^large^	<.001^large^	<.001^trivial^
		W_ANA_ (kJ)^a^	223.4 ± 37.1	69.0 ± 14.9	68.3 ± 14.7	<.001^large^	<.001^large^	<.001^large^	<.001^trivial^

Means, standard deviations, *p*-values, and interpretations of effect sizes (superscripted) are shown.

COD, change of direction; fix, fixed energy equivalent; ind, individual energy equivalent; MPM, metabolic power model; W_AER_, aerobic energy supply; W_ANA_, anaerobic alactic and lactic energy supply; W_TOT_, total energy expenditure.

^a^ Analyzed by one-way repeated measures ANOVA and *t*-test.

^b^ Analyzed by Friedman and Wilcoxon test.

**Figure 6:**
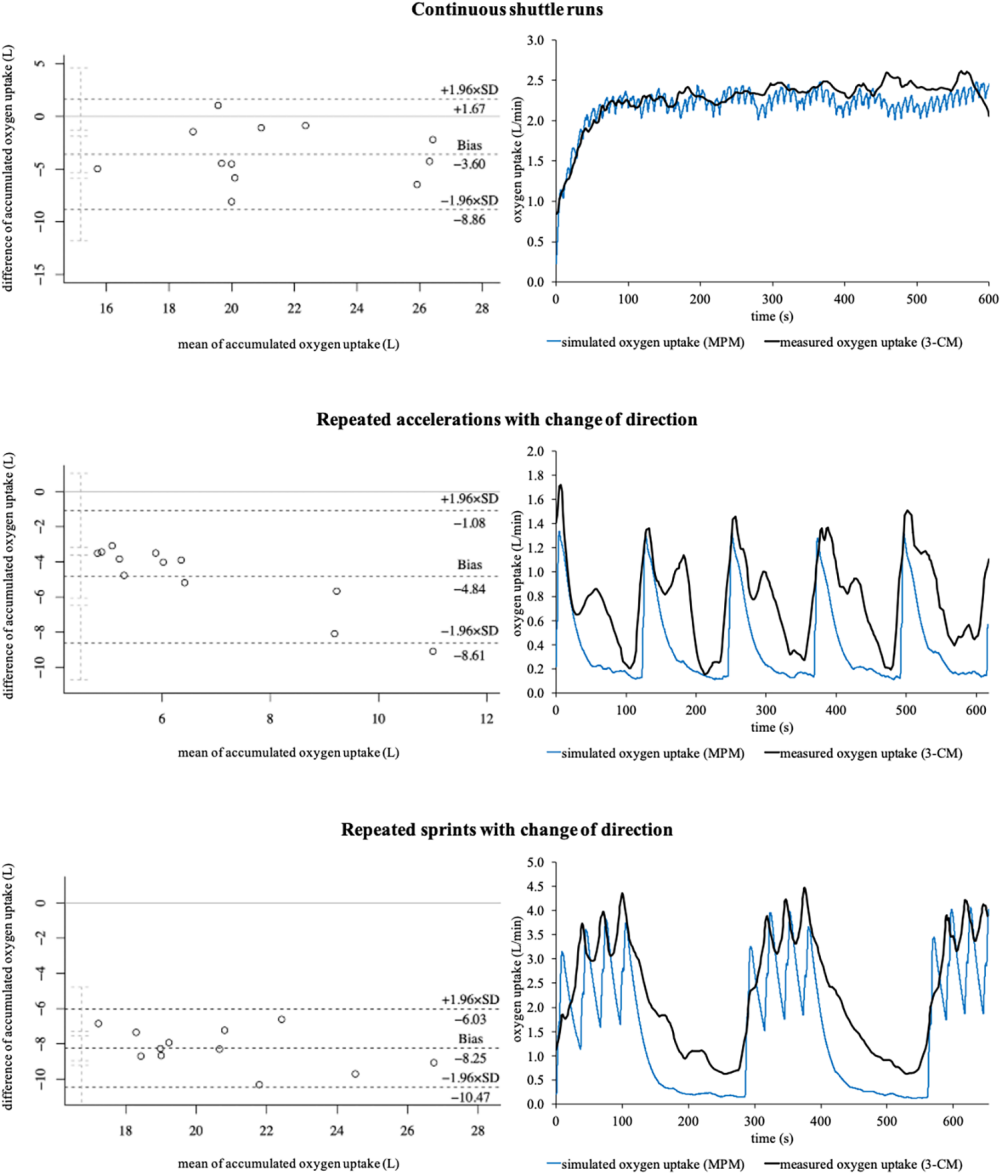
Bland-Altman plots of the accumulated oxygen uptakes and sample courses of the simulated (metabolic power model) and measured oxygen uptakes (3-component model) of the three running-based exercises. 3-CM, 3-component model; MPM, metabolic power model; SD, standard deviation.

Regarding accumulated oxygen uptake, statistically significant differences of *p* ≤ .001 (*d* ≥ 1.002, large) were detected for all three running-based exercises. Figure 6 shows the corresponding Bland-Altman plots as well as sample courses of the simulated and measured oxygen uptakes of the three exercises. The mean relative biases for the metabolic power model were −15.2 ± 11.6%, −53.0 ± 5.8%, and −33.4 ± 3.8% for the continuous shuttle runs, repeated accelerations, and repeated sprints, respectively.

## Discussion

As far as we know, this is the first study to validate the metabolic power model against the established 3-component model allowing direct comparison of variables including total energy expenditure and aerobic and anaerobic supply during three intermittent running-based exercises. The main findings were that the metabolic power model significantly under- or overestimates (i) total energy expenditure and (ii) energy supplies with large effect sizes, particularly in intermittent exercises.

In preparation for validating the metabolic power model, we generated three different running-based exercises intended to primarily focus on one of the three main energy supplies. As seen in Table 1, the effects on the external and internal loads are mostly statistically significantly different from each other, confirming our intention. In line with this, and based on the 3-component model, the energy supplies during the three running-based exercises clearly differ (Figure 4). This is especially true when excluding the passive breaks between efforts. Even though the aerobic energy supply still has the highest contribution in all three exercises (≥61.3%), the intended activation of the two anaerobic pathways is given, with contributions of 20.2% for the anaerobic alactic and 35.0% for the lactic energy supply regarding the repeated accelerations and repeated sprints, respectively. Additionally, we investigated the influence of using an individual or fixed energy equivalent of oxygen for the 3-component model. However, while our results showed significant differences, the effects were trivial (Table 2). Therefore, individualized energy equivalents might be neglected, in favor of the fixed energy equivalent of 20.9 kJ/L O2, which is already implemented in the metabolic power model (18). Furthermore, our data revealed a large influence regarding the inclusion or exclusion of the passive breaks (Table 2). This supports our research design to control the resulting bias and allowed us to specify if the potential bias concerning the metabolic power model is dependent on the type of energy supply. In this context, however, it is worth mentioning that a modified “intermittent” 3-component model has been developed taking the elevated oxygen uptake used to replenish phosphocreatine during passive breaks into account (52). Although this intermittent model might offer a promising approach, we decided to use the established 3-component model for two reasons. Firstly, it has been applied more often and is clearly better evaluated in the scientific literature yet (53, 54). Especially, a recent study showed a superior reliability in terms of aerobic (CV: 3.62% vs. 6.04%) and anaerobic energy supply (CV: 7.49% vs. 8.95%) (55). Secondly, since previous validation studies neglected the different approaches of the 3-component model and metabolic power model during passive breaks (elevated oxygen uptake vs. no data generation) (28, 29, 31, 32), we intended to control for this possible bias, which is not possible in the intermittent 3-component model, without modifying its basic assumptions and calculations. However, for the readers and future research, the differences between both 3-component models and the metabolic power model during the repeated acceleration and sprint exercises including the elevated oxygen uptake during the passive breaks can be found in the supplementary material (Supplementary Table 1).

Our first main finding showed that the metabolic power model significantly under- or overestimates the total energy expenditure with large effect sizes during continuous shuttle runs (−13.5 ± 11.8%), repeated accelerations (352.2 ± 115.9%), and sprints (75.0 ± 17.0%) (Table 2, Figure 5). This partly confirms findings from previous studies, where e.g., energy expenditure was up to 66% lower during team-sport-specific circuits when measured with the metabolic power model compared to that derived through portable respiratory gas analyzers (31, 56). However, it is important to notice that these circuits included activities, which are unable to be tracked by the metabolic power model, such as jumps, collision, or technical skills such as ball handling. Ulupinar et al. (57) investigated 18 male university league soccer players during two different repeated sprint protocols, also using the 3-component model. In this study, absolute total energy expenditures of 586.3 ± 60.8 kJ and 595.6 ± 57.5 kJ for 10 × 40 m and 20 × 20 m sprints were reported, respectively. These values support our results of the repeated sprints, but only when including the passive breaks (586.6 ± 74.2 kJ). Without the breaks, values were significantly lower due to the aerobic energy supply (174.7 ± 22.1 kJ) (Table 2). Regarding the metabolic power model and its specific calculations, some aspects need to be considered that may explain the observed differences between the two models. Firstly, the model assumes that the relative energy cost is independent of speed (4, 13, 15). This, however, can only be accepted to a limited extent, as it has been shown that energy cost during shuttle running is approximately 30–50% higher compared to constant speed running and this discrepancy increases with increasing speed (30). Secondly, the equivalent slope model, on which the calculations are based, has only been developed up to a slope of .45° (15) corresponding to an acceleration of 4.5 m/s^2^ (4). Any slopes above this threshold must be extrapolated; however, mean peak accelerations in our study were up to 4.25 m/s^2^ (Table 1) and thus still within the range of the equivalent slope model. Lastly, the ability of global positioning devices to validly record accelerations, the use of respective filtering techniques for speed and acceleration data, and the influence of different surfaces and footwear must be taken into account (19). The specific reasons for the significant under- or overestimation of the metabolic power model in terms of total energy expenditure cannot be definitively identified here, but the calculation aspects listed may have an influence on the results, requiring more research.

Our second main finding showed that the aerobic energy supply is significantly under- but anaerobic energy supply overestimated by the metabolic power model with large effect sizes regardless of the exercise condition (Table 2, Figure 5). This is especially true regarding the anaerobic energy supply with biases of up to 1,849.9 ± 831.8%. To the best of our knowledge, there are currently no other studies that have investigated the energy supplies by the metabolic power model in intermittent sports. However, as the estimation of the aerobic and anaerobic energy supply results from the time courses of simulated oxygen uptake and metabolic power by that model, the accumulated oxygen uptakes and respective kinetics of the measured (3-component model) and simulated oxygen uptakes (metabolic power model) were compared (Figure 6), which may explain the differences. The metabolic power model revealed relative biases of up to −53.0 ± 5.8% for the accumulated oxygen uptake. While the bias was lowest during the continuous shuttle runs and therefore mainly aerobic energy supply, biases increased when the anaerobic energy supplies were more heavily stressed (Figure 6). Concerning the continuous shuttle runs, the simulated oxygen uptake shows a rather physiological progression (Figure 6), as described before (58). Regarding the repeated accelerations and sprints with change of direction, the simulated oxygen uptake does not follow a physiological time course, especially during the offset periods, where too steep descends can be seen, as opposed to a typical course (41). During the onset periods, there are slightly delayed responses combined with too steep ascends (Figure 6). These observations indicate that the increasing bias may be due to the less physiological time courses of the simulated oxygen uptake. Additionally, the metabolic power model assumes that the aerobic supply is the proportion below the simulated oxygen uptake, but only if it is simultaneously below the metabolic power (18). While this may be appropriate for estimating the energy expenditure of running only, the overall energy expenditure of exercises (i.e., of the entire body) may require to take the total proportion below the simulated oxygen uptake independent of the time course of metabolic power into account. Therefore, the simulation of oxygen uptake and calculation of aerobic supply by the metabolic power model should be reconsidered and potentially adapted accordingly. This in turn may positively influence the estimation of the aerobic and anaerobic supplies in future studies.

From a practical point of view, the metabolic power model may be considered as a promising new approach to investigate metabolic loads in intermittent sports during training and matches when it is further optimized by future studies. At this time, its use should be treated with caution, because our results show large effect sizes and biases compared to the established 3-component model (Table 2; Figure 5). These outcomes indicate that the metabolic power model may be systematically flawed for certain exercises, especially those requiring high anaerobic energy supply. For practical applications, this is a problem due to the fact that many activities being associated with the playing success such as maximum accelerations and sprints require a high anaerobic supply in intermittent sports (59) and the amount of anaerobic supply also affects the time required to recover after training and matches (60, 61). This issue might lead to misinterpretation of the actual metabolic load during competitions and consequently incorrect training and recovery prescriptions. A further practical challenge is that the model is incapable to differentiate between anaerobic alactic and lactic energy supply (18) as well as to validly capture elevated oxygen uptake during passive breaks being crucial for the replenishment of phosphocreatine yet (Figure 6). Since energy systems shift rapidly during intermittent sports, more research is required to optimize the metabolic power model for allowing more specific training and recovery prescriptions. In this context, one further possibility may be to incorporate heart rate for improving the simulation of oxygen uptake (62) or blood lactate data to possibly help differentiate between anaerobic alactic and lactic energy supply (4) during passive breaks into the model's estimates. Although the assessment of these data is not always allowed and accepted by the athletes during competitive matches, it may be worth considering for training purposes where the assessment could be taken into account, requiring more research.

While our study clearly increased the knowledge on the metabolic power in intermittent sports, few limitations exist. Firstly, we only investigated male trained athletes participating three to four times per week in their intermittent sport. Thus, our results cannot be generalized to females and children or other subpopulations with different physical prerequisites or activity levels for which future studies, taking these characteristics into account, are required. Secondly, although we noted weather conditions (i.e., temperature, wind speed, air humidity), we did not investigate how they might have affected our results. Thirdly, regarding the Interval Shuttle Run Test to assess maximum oxygen uptake, we considered three established criteria to clarify exhaustion. However, the validity of these and further criteria (e.g., respiratory exchange ratio) is still controversially discussed (63). Fourthly, we were only able to correct the passive breaks for the aerobic but not the anaerobic energy supply, which may have an influence regarding the discrepancies of the reported anaerobic energy supplies between the two models. Lastly, though not directedly related to the weakness of our study, we used the 3-component model as an established standard in exercise science to validate the metabolic power model, which, however, also has flaws: Contrary to the metabolic power model, the 3-component model only allowed us to analyze the total exercise, not each individual effort, which would have enabled an even better comparison. Moreover, while the reliability of the 3-component model has been reported for the aerobic (CV = 3.62%), anaerobic alactic (CV = 14.85%), and anaerobic lactic (CV = 11.43%) contribution (55), its validity is still under discussion and there are no corresponding statistical indices available yet. A main reason is that there is no established method to directedly and independently access both anaerobic supplies (64), which is also indicated by physiological discrepancies. For example, concerning the anaerobic alactic supply, it is questionable, how the mathematical fitting of the fast component of the excess post-exercise oxygen consumption should be conducted (65). Furthermore, it is unknown, if there is an independence of the lactate removal from the blood during this post-exercise period (54, 64). Regarding the anaerobic lactic supply, repeated invasive lactate analyses need to be carried out, for which reproducibility of the absolute values is questionable (66) and it is also still uncertain, whether the commonly used oxygen lactate equivalent of 3 ml O_2_/kg is appropriate for all individuals (50, 67). Noteworthy, and as mentioned above, a modified “intermittent” 3-component model has been developed during the last years providing a promising comparative alternative (52). As explained before, we decided to use the established 3-component model. For interested readers and future research, we present the differences between the two 3-component-models and the metabolic power model in the supporting information (Suppplementary Table S1). Although, the comparison of the two 3-component models showed statistically significant differences, the comparison of both models to the metabolic power model showed the same statistical outcomes with large effect sizes. Taken together, all these findings underline the lasting need to optimize both 3-component models and the metabolic power model for assessing metabolic loads during intermittent exercises (68).

## Conclusion

In conclusion, this study showed that the metabolic power model significantly under- or overestimates total energy expenditure and energy supplies with large effect sizes during intermittent running-based exercises. The reason may be due to the calculation of metabolic power and simulated oxygen uptake themself as well as the unphysiological time courses of simulated oxygen uptake in particular. Future studies should optimize these points with a special focus on the improvement of the simulated oxygen uptake kinetics. Until these issues can be fixed, the use of the metabolic power model should be handled with caution, especially in scientific and practical purposes as it might lead to misinterpretation of the data and thus incorrect training and recovery prescriptions.

## Data Availability

The original contributions presented in the study are included in the article/Supplementary Material, further inquiries can be directed to the corresponding author.
