# Supplementary material for: Validation of the metabolic power model during three intermittent running-based exercises with emphasis on aerobic and anaerobic energy supply
Source: Front Sports Act Living. 2025 Apr 17;7:1583313. doi: 10.3389/fspor.2025.1583313 (PMC12043615; doi:10.3389/fspor.2025.1583313)
Supplement: Supplementary file 1 [file Table1.docx]

Supplementary Table

**Table 1.** Differences in total energy expenditure and energy supplies of the repeated accelerations and sprints with change of direction using the metabolic power model, the 3-component model, and the intermittent 3-component model with a fixed energy equivalent.

| Running-based exercises | Variables | Metabolic power model  mean ± SD | 3-component model  mean ± SD | Intermittent  3-component model  mean ± SD | Global  p-value | MPM vs. 3CM  p-value | MPM vs. 3CM_int_  p-value | 3CM vs. 3CM_int_  p-value |
| --- | --- | --- | --- | --- | --- | --- | --- | --- |
| Repeated accelerations with COD | W_TOT_ (kJ)^#^ | 92.6 ± 25.4 | 193.4 ± 63.3 | 193.4 ± 63.3 | <.001 ^large^ | <.001 ^large^ | <.001 ^large^ | / |
|  | W_AER_ (kJ^)#^ | 41.8 ± 15.1 | 188.8 ± 61.8 | 176.0 ± 59.8 | <.001 ^large^ | <.001 ^large^ | <.001 ^large^ | <.001 ^small^ |
|  | W_ANA_ (kJ)^#^ | 50.8 ± 11.4 | 4.6 ± 1.9 | 17.4 ± 5.9 | <.001 ^large^ | <.001 ^large^ | <.001 ^large^ | <.001 ^large^ |
| Repeated sprints  with COD | W_TOT_ (kJ)^*^ | 376.1 ± 62.1 | 586.6 ± 74.2 | 586.6 ± 74.2 | <.001 ^large^ | <.001 ^large^ | <.001 ^large^ | / |
|  | W_AER_ (kJ)^*^ | 152.7 ± 26.5 | 518.3 ± 64.7 | 451.4 ± 59.7 | <.001 ^large^ | <.001 ^large^ | <.001 ^large^ | <.001 ^large^ |
|  | W_ANA_ (kJ)^*^ | 223.4 ± 37.1 | 68.3 ± 14.7 | 135.3 ± 25.4 | <.001 ^large^ | <.001 ^large^ | <.001 ^large^ | <.001 ^large^ |
| Note: Means, standard deviations, p-values, and interpretations of effect sizes (superscripted) are shown. ^*^ = analyzed by one-way repeated measures ANOVA and t-test; ^#^ = analyzed by Friedman and Wilcoxon test; 3CM = 3-component model; 3CM_int_ = intermittent 3-component model; COD = change of direction; MPM = metabolic power model; W_AER_ = aerobic energy supply; W_ANA_ = anaerobic alactic and lactic energy supply; W_TOT_ = total energy expenditure. | | | | | | | | |
